# Diagnostic Accuracy of Non-Contrast CT for Acute Appendicitis in the Emergency Department: A Systematic Review and Meta-Analysis

**DOI:** 10.3390/medicina61122163

**Published:** 2025-12-04

**Authors:** Se Kwang Oh

**Affiliations:** 1 Department of Emergency Medicine, Chungnam National University Sejong Hospital, Sejong 30099, Republic of Korea; 13744@hanmail.net or skoho@cnuh.co.kr; Tel.: +82-44-995-3021; 2 Department of Emergency Medicine, College of Medicine, Chungnam National University, Daejeon 34131, Republic of Korea

**Keywords:** appendicitis, tomography, X-ray computed, sensitivity and specificity, emergency service, hospital, meta-analysis as topic

## Abstract

*Background and Objectives:* Contrast-enhanced computed tomography (CT) is widely regarded as the gold standard for diagnosing acute appendicitis. However, the use of contrast agents may be contraindicated in patients with renal impairment or a history of allergic reactions. Non-contrast CT (NCCT) offers a potential alternative, but its diagnostic performance has been variably reported across studies. This systematic review and meta-analysis aimed to evaluate the pooled diagnostic accuracy of NCCT in detecting acute appendicitis. *Materials and Methods:* A comprehensive literature search was conducted across PubMed, Ovid MEDLINE, EMBASE, Cochrane Library, and Google Scholar from inception to June 2025. Studies assessing the diagnostic accuracy of NCCT for acute appendicitis were included. Pooled sensitivity, specificity, and area under the hierarchical summary receiver operating characteristic (HSROC) curve were estimated using a bivariate random-effects model. Study quality was assessed with the QUADAS-2 tool, and publication bias was evaluated using Deeks’ funnel plot asymmetry test. *Results:* Eleven studies comprising 1996 patients met the inclusion criteria. The pooled sensitivity and specificity of NCCT were 0.93 (95% confidence interval; CI, 0.91–0.95) and 0.97 (95% CI, 0.95–0.97), respectively. The area under the HSROC curve was 0.89 (95% CI, 0.83–0.96), indicating moderate diagnostic performance. Heterogeneity was moderate for sensitivity (I^2^ = 48.2%) and substantial for specificity (I^2^ = 77.6%), likely due to differences in study populations and CT acquisition protocols. No significant publication bias was detected (Deeks’ test, *p* = 0.079). *Conclusions:* NCCT demonstrates moderate diagnostic accuracy for detecting acute appendicitis and offers a practical alternative for patients who cannot receive contrast media. Its safety and rapid applicability make NCCT a useful imaging option in emergency settings, especially when contrast use is limited.

## 1. Introduction

Acute appendicitis remains one of the most common causes of abdominal pain requiring surgical intervention in emergency departments worldwide [1,2,3]. Prompt and precise diagnosis is essential to reduce the risk of complications, including perforation, abscess formation, and sepsis. Computed tomography (CT) has been widely recognized as a reliable imaging tool for the diagnosis of acute appendicitis, with consistently high sensitivity and specificity [4,5,6].

CT is a valuable modality in the diagnosis of acute appendicitis; however, its reliance on contrast agents can pose limitations, particularly in patients with impaired renal function, a prior history of allergic or other contrast-related reactions, or those who are clinically unstable [7,8,9]. As alternatives, ultrasonography and magnetic resonance imaging (MRI) have been extensively studied for their diagnostic accuracy in acute appendicitis. In addition, several studies have specifically assessed the diagnostic performance of non-contrast computed tomography (NCCT), providing additional evidence regarding its potential clinical utility [10,11,12,13,14,15,16,17,18,19,20,21,22].

In addition to imaging, several clinical scoring systems and inflammatory biomarkers are frequently used to support the diagnostic assessment of patients with suspected acute appendicitis. Tools such as the Alvarado score, the Appendicitis Inflammatory Response (AIR) score, and the Adult Appendicitis Score (AAS) help stratify patients by integrating key symptoms, physical findings, and basic laboratory results, thereby guiding early clinical decision-making [23,24,25,26,27]. The Alvarado score is widely used in the initial evaluation of patients with suspected appendicitis; however, it has several well-recognized limitations, including reduced accuracy in women and older adults, the absence of key inflammatory biomarkers, and limited ability to reliably confirm or exclude appendicitis, particularly in intermediate-risk patients [24,28,29].

Although the AIR and AAS scores are useful for identifying patients in the low-risk category, they do not provide sufficient diagnostic accuracy to confidently confirm or rule out appendicitis in intermediate- or high-risk groups. Even though the AIR score incorporates objective inflammatory markers and generally outperforms the Alvarado score, it still lacks the reliability needed to serve as a standalone diagnostic tool, making imaging essential for establishing a definitive diagnosis in most clinical scenarios [27,30,31].

Similarly, inflammatory biomarkers such as leukocytosis, C-reactive protein (CRP), and procalcitonin may offer supplementary diagnostic clues in patients with suspected appendicitis; however, these tests show considerable variability in accuracy, and neither CRP levels nor leukocyte counts can reliably confirm or exclude appendicitis, with sensitivities and specificities generally lower than those achieved with CT [32,33,34,35,36,37]. More recently, salivary biomarkers have been investigated as a noninvasive adjunct, offering simple collection and minimal patient discomfort, especially in children, but they still require further validation [38,39,40].

Although clinical scoring systems and inflammatory biomarkers can aid in the diagnostic assessment of acute appendicitis, their inherent limitations reduce their reliability. Consequently, CT remains essential for establishing a definitive diagnosis in emergency settings.

NCCT provides practical advantages, including rapid acquisition, broad availability, and the avoidance of contrast-related risks. Nevertheless, uncertainty persists regarding its diagnostic performance relative to contrast-enhanced CT in the evaluation of acute appendicitis. Several previous studies examining the diagnostic accuracy of NCCT for acute appendicitis have yielded heterogeneous results, highlighting the need for evidence synthesis. Accordingly, we conducted a systematic review and meta-analysis to evaluate the diagnostic performance of NCCT for acute appendicitis in patients presenting to emergency departments.

## 2. Materials and Methods

### 2.1. Study Design

This systematic review and meta-analysis was designed and conducted to evaluate the diagnostic accuracy of NCCT in detecting acute appendicitis. The study adhered to the Preferred Reporting Items for Systematic Reviews and Meta-Analyses (PRISMA) 2020 statement to ensure methodological rigor and transparency [41].

A pre-specified protocol, outlining the study objectives, inclusion criteria, and analytical methods, was developed prior to data collection and registered in the International Prospective Register of Systematic Reviews (PROSPERO; registration number: CRD420251117455; registered on 1 August 2025).

### 2.2. Data Sources and Search Strategy

A comprehensive literature search was conducted to identify relevant studies evaluating the diagnostic performance of NCCT in detecting acute appendicitis. Five major electronic databases—PubMed, Ovid MEDLINE, EMBASE, the Cochrane Library, and Google Scholar—were systematically searched from their inception to June 2025. Both Medical Subject Headings (MeSH) terms and free-text keywords were used in combination with Boolean operators. The core search expression was as follows: (“appendicitis” OR “acute appendicitis”) AND (“non-contrast CT” OR “non-enhanced computed tomography” OR “unenhanced CT” OR “helical CT” OR “spiral CT”) AND (“diagnostic accuracy” OR “sensitivity” OR “specificity” OR “diagnosis”) (Supplementary Table S1). Only studies conducted on human participants and published in English were considered eligible, with no restrictions on publication year or study design.

Two independent reviewers (S.K.O. and S.U.C.) conducted the study screening process. Both reviewers independently performed title and abstract screening, followed by full-text assessments for studies meeting the initial criteria. Any disagreements were resolved through discussion and consensus. The final database search was completed on 10 June 2025. The study selection process adhered to PRISMA guidelines and is summarized in the PRISMA flow diagram.

### 2.3. Eligibility Criteria and Study Selection

Studies were considered eligible if they met the following predefined inclusion criteria. Original studies that assessed the diagnostic accuracy of NCCT for identifying acute appendicitis were included. Eligible studies enrolled patients presenting with clinically suspected appendicitis, most commonly in emergency department settings, and reported diagnostic performance measures such as sensitivity and specificity, or provided sufficient raw data to construct a 2 × 2 diagnostic contingency table (true positives, false positives, true negatives, and false negatives). The diagnostic reference standard was required to include surgical or histopathological confirmation for positive cases and clinical follow-up or imaging confirmation for negative cases. Studies employing prospective or retrospective observational designs were both considered appropriate for inclusion.

Studies were excluded if they used contrast-enhanced CT alone or were case reports, editorials, conference abstracts, or narrative reviews. Research that did not provide adequate data for calculating diagnostic accuracy indices was also excluded. Study selection was performed through a two-step process. Initially, two independent reviewers screened the titles and abstracts of all retrieved records to remove irrelevant studies. Subsequently, potentially eligible articles underwent full-text evaluation to confirm inclusion. Any disagreements between reviewers were resolved through discussion, and a final decision was reached by consensus.

### 2.4. Quality Assessment and Risk of Bias Evaluation

The methodological quality of the included studies was appraised using the Quality Assessment of Diagnostic Accuracy Studies-2 (QUADAS-2) framework [42]. This structured approach examines potential bias and applicability issues within four core domains: patient selection, index test, reference standard, and flow and timing. The detailed signaling questions and assessment criteria used for each domain are summarized in Supplementary Table S2. Each domain was systematically reviewed based on the signaling questions provided in the QUADAS-2 manual, and the degree of bias and applicability concern was judged as low, high, or unclear. To maintain objectivity, two reviewers independently assessed the methodological quality of all eligible studies. Any disagreements between reviewers were discussed, and consensus was reached. The level of inter-rater agreement was calculated using Cohen’s kappa statistic, providing a quantitative measure of consistency between assessments. A comprehensive overview of study quality was generated, and potential sources of bias were illustrated through a risk-of-bias figure and summary table.

### 2.5. Statistical Analysis and Data Synthesis

Data extracted from each included study comprised information on study design, sample size, and diagnostic performance measures such as sensitivity, specificity, positive predictive value (PPV), and negative predictive value (NPV). To summarize the diagnostic accuracy of non-contrast computed tomography (NCCT) for acute appendicitis, a bivariate random-effects meta-analysis was performed. This model allowed the concurrent estimation of sensitivity and specificity while taking into account variability both within and between studies. Such an approach was deemed suitable for addressing potential clinical and methodological heterogeneity among the included studies. The extent of heterogeneity was assessed using the I^2^ statistic and Cochran’s Q test, with higher I^2^ values indicating greater inconsistency. Forest plots were constructed to display individual study estimates of sensitivity and specificity, providing a visual overview of diagnostic performance. In addition, a hierarchical summary receiver operating characteristic (HSROC) model, fitted using the bivariate random-effects approach (Reitsma method), was used to further evaluate the overall discriminative ability of NCCT. This model jointly estimated logit-transformed sensitivity and specificity, considering study-level variances. The HSROC curve and the corresponding summary operating point were derived from the model, and the area under the curve (AUC) was computed by numerical integration. The 95% confidence interval of the AUC was determined through nonparametric bootstrapping with 2000 replications.

After model fitting and summary estimation, potential publication bias was assessed using Deeks’ funnel plot asymmetry test. All statistical analyses were performed using Meta-DiSc (version 1.4), Review Manager (RevMan, version 5.3), STATA (version 17.0; StataCorp, College Station, TX, USA), and the metaDTA online application (https://crsu.shinyapps.io/metaDTA/, accessed on 10 September 2025).

## 3. Results

### 3.1. Search Results and Study Selection Flow

**Figure 1:**
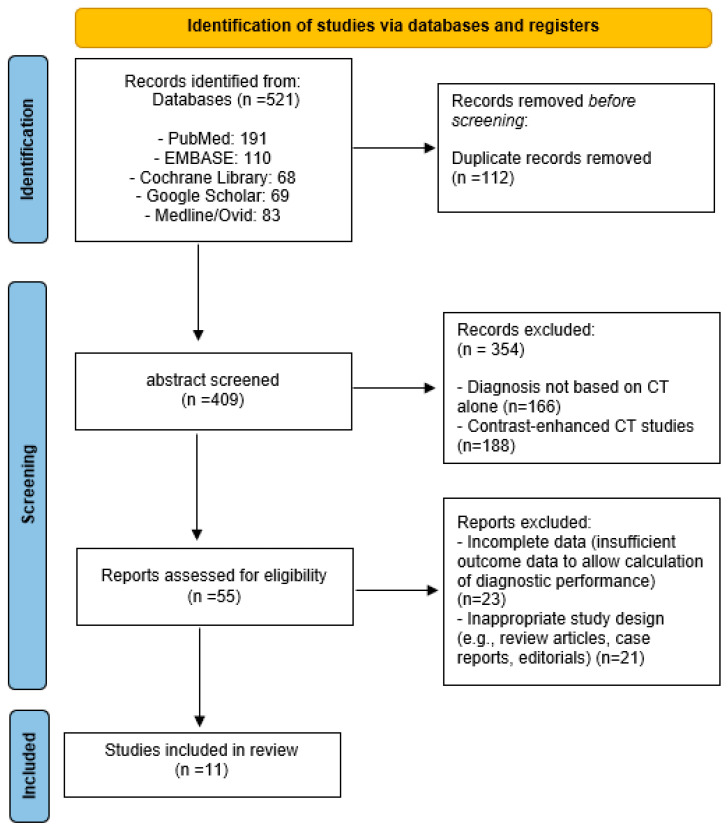
PRISMA flow diagram of study selection process.

A total of 521 records were initially identified through electronic database searches, comprising PubMed (191), EMBASE (110), Cochrane Library (68), Google Scholar (69), and Ovid MEDLINE (83). After eliminating 112 duplicates, 409 unique articles remained for title and abstract screening. At this stage, 354 studies were excluded because they did not meet the inclusion criteria—specifically, 166 studies that did not rely solely on CT for diagnosis and 188 that used contrast-enhanced CT protocols. The full texts of 55 potentially eligible articles were subsequently reviewed in detail. Of these, 23 studies lacked sufficient diagnostic data to calculate performance indices such as sensitivity or specificity, and 21 were excluded owing to inappropriate design, including review articles, case reports, or editorials. Ultimately, 11 studies fulfilled all eligibility criteria and were incorporated into the final systematic review and meta-analysis (Figure 1). The main characteristics of the included studies, including publication year, study design, patient population, and diagnostic performance metrics, are summarized in Table 1.

### 3.2. Assessment of Methodological Quality and Risk of Bias

The methodological quality of the included studies was evaluated using the Quality Assessment of Diagnostic Accuracy Studies-2 (QUADAS-2) tool. Two reviewers (S.K. Oh and S.U. Cho) independently assessed each study for potential risk of bias and applicability concerns, and any disagreements were resolved through discussion.

**Figure 2:**
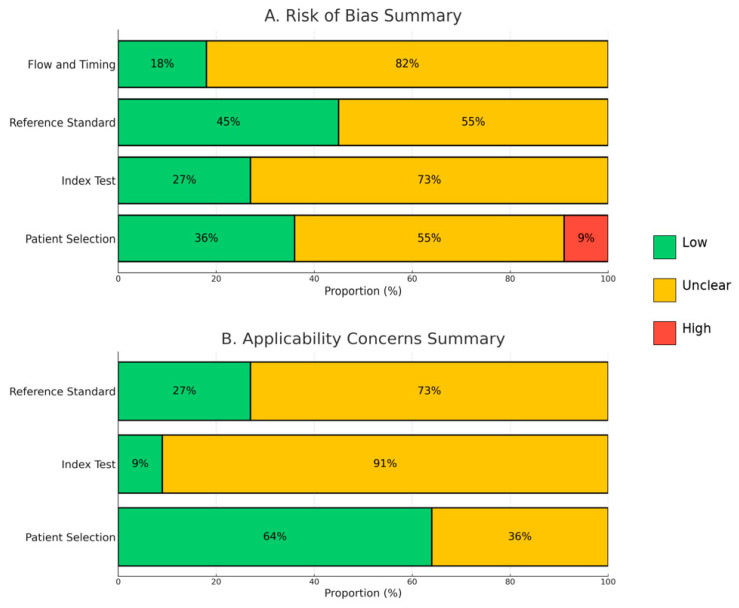
Summary of methodological quality assessment using the QUADAS-2 tool.

Figure 2 summarizes the overall results of the QUADAS-2 assessment. In general, no major sources of bias were identified, although several domains demonstrated some degree of uncertainty. The Index Test, Reference Standard, and Flow and Timing domains were frequently rated as unclear, mainly because of incomplete reporting of CT protocols, interpretation criteria, or patient flow between testing steps. The Patient Selection domain exhibited a mixed pattern, with a notable proportion of studies rated as unclear or high risk, suggesting that inclusion and exclusion criteria were not always clearly described. A numerical summary of all QUADAS-2 ratings has been added in Supplementary Table S3 to complement the graphical presentation.

**Figure 3:**
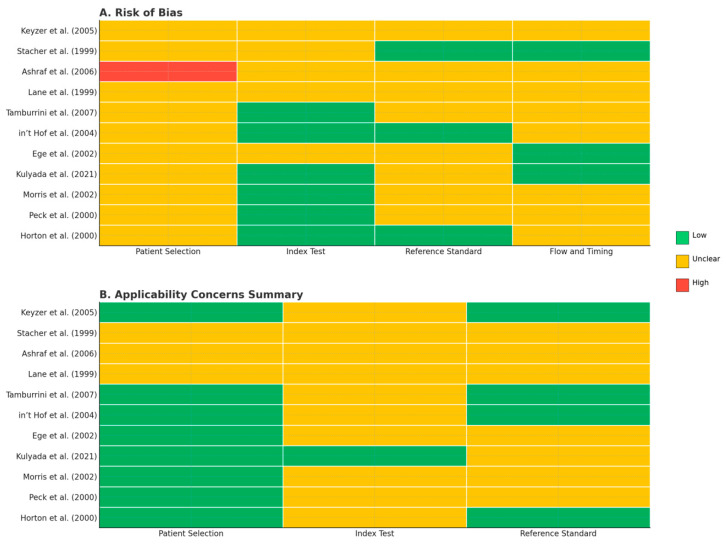
Traffic-light plots for methodological quality assessment using the QUADAS-2 tool. Panel (**A**) illustrates the risk-of-bias evaluation, and Panel (**B**) presents the applicability assessment. Each domain is color-coded as low risk (green), unclear risk (yellow), or high risk (red) [12,13,14,15,16,17,18,19,20,21,22].

Figure 3 provides a detailed “traffic-light” visualization of individual study assessments. Panel (A) illustrates the risk-of-bias evaluation, and Panel (B) presents the applicability assessment, with color coding indicating low (green), unclear (yellow), or high (red) risk levels. Overall, the included studies showed minimal risk of bias, though incomplete methodological descriptions in a few domains introduced some uncertainty.

Bar charts summarizing the overall risk of bias (A) and applicability concerns (B) across all included studies. Each domain is expressed as the percentage of studies rated as low risk (green), unclear risk (yellow), or high risk (red).

### 3.3. Pooled Diagnostic Accuracy and Performance Analysis

#### 3.3.1. Pooled Estimates (Primary Analysis)

**Figure 4:**
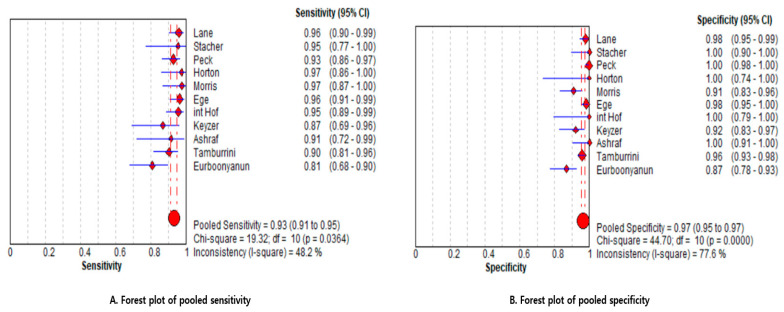
Forest plots of pooled sensitivity and specificity for non-contrast computed tomography in diagnosing acute appendicitis. (**A**) Forest plot showing individual and pooled estimates of sensitivity with 95% confidence intervals. (**B**) Forest plot showing individual and pooled estimates of specificity with 95% confidence intervals [12,13,14,15,16,17,18,19,20,21,22].

As shown in the forest plots (Figure 4), the included studies consistently demonstrated reliable diagnostic performance of NCCT in evaluating acute appendicitis. Using a bivariate random-effects meta-analysis, the pooled sensitivity was estimated at 0.93 (95% CI, 0.91–0.95) and the pooled specificity at 0.97 (95% CI, 0.95–0.97). Across individual studies, sensitivity values ranged from 0.81 to 0.97, while specificity varied between 0.87 and 1.00. These results suggest that NCCT is a reliable imaging modality for diagnosing acute appendicitis in emergency care settings.

#### 3.3.2. Sensitivity Analysis Based on Risk of Bias

To assess the robustness of the pooled estimates, we conducted a sensitivity analysis excluding the single study rated as high risk in the Patient Selection domain [20]. The pooled sensitivity and specificity remained virtually unchanged compared with the primary analysis (sensitivity: 0.931 vs. 0.932; specificity: 0.965 vs. 0.964), indicating that the overall diagnostic performance of NCCT was stable and not materially influenced by the exclusion of this study.

#### 3.3.3. Between-Study Heterogeneity

As shown in Figure 4, statistical heterogeneity was moderate for sensitivity and substantial for specificity, with I^2^ = 48.2% for sensitivity (Cochran’s Q = 19.32; df = 10; *p* = 0.0364) and I^2^ = 77.6% for specificity (Q = 44.70; df = 10; *p* < 0.001), respectively. This variability may be explained by differences in diagnostic thresholds, reference standards, or patient characteristics across studies, with heterogeneity more evident in specificity.

#### 3.3.4. Summary ROC (HSROC) Model

**Figure 5:**
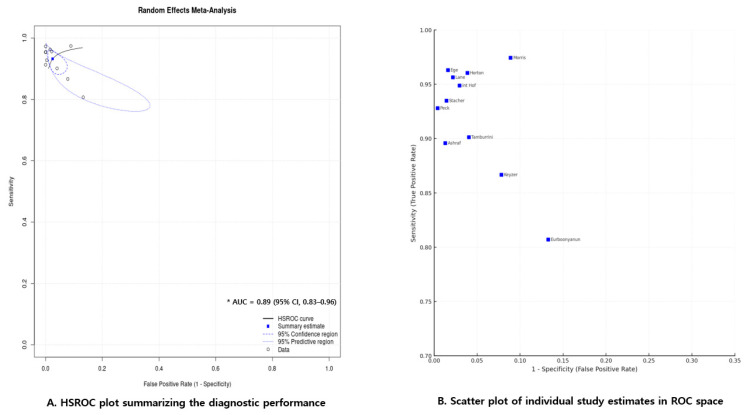
Summary plots of diagnostic accuracy meta-analysis. (**A**) Hierarchical summary receiver operating characteristic (HSROC) plot showing the pooled diagnostic performance of non-contrast computed tomography for acute appendicitis. The summary estimate, 95% confidence region, and 95% prediction region are shown, with an area under the curve (AUC) of 0.89 (95% CI, 0.83–0.96). (**B**) Scatter plot of individual study estimates in receiver operating characteristic (ROC) space, illustrating the distribution of sensitivity and false-positive rate across the included studies [12,13,14,15,16,17,18,19,20,21,22].

To further evaluate the diagnostic performance of NCCT, a hierarchical summary receiver operating characteristic (HSROC) model was fitted using the bivariate random-effects framework proposed by Reitsma et al. [43]. This approach allowed for the simultaneous estimation of sensitivity and specificity while accounting for study-level heterogeneity and the correlation between measures. The fitted model yielded an area under the curve (AUC) of 0.89 (95% CI, 0.83–0.96), indicating that NCCT has moderate discriminative ability in distinguishing acute appendicitis from non-appendicitis cases (Figure 5A). The summary point estimated from the HSROC model showed close agreement with the pooled results, indicating that the overall model provided consistent and dependable estimates across the included studies. In addition to the primary HSROC estimates, we calculated the between-study variance (τ^2^) and the correlation between logit-transformed sensitivity and specificity to improve transparency and reproducibility. The τ^2^ values were 0.21 for sensitivity and 0.58 for specificity, indicating greater heterogeneity in specificity. The estimated correlation coefficient (r = 0.31) suggested only a modest relationship between sensitivity and specificity across studies.

#### 3.3.5. Study-Level Distribution of Diagnostic Estimates in ROC Space

The scatter plot of individual studies in ROC space (Figure 5B) shows that most data points cluster toward the upper-left quadrant, reflecting consistently reliable sensitivity and specificity in most studies. A few outlying studies with lower values in either metric likely contributed to the observed heterogeneity, as illustrated by the predictive region of the HSROC curve.

#### 3.3.6. Publication Bias Assessment

**Figure 6:**
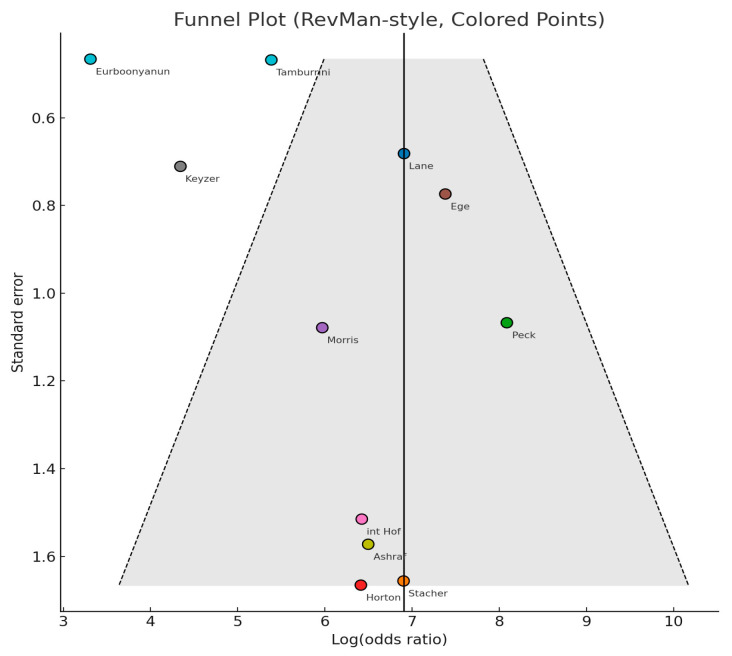
Funnel plot assessing publication bias among studies evaluating the diagnostic accuracy of non-contrast computed tomography for acute appendicitis. Each dot represents an individual study plotted by its log odds ratio and standard error. The funnel shape appears largely symmetrical, indicating no significant publication bias (Egger’s test, *p* = 0.079) [12,13,14,15,16,17,18,19,20,21,22].

The possibility of publication bias was evaluated using Deeks’ funnel plot asymmetry test. As shown in Figure 6, the funnel plot was generally symmetrical upon visual inspection, suggesting no apparent indication of publication bias. Egger’s regression analysis produced a *p*-value of 0.079, implying that small-study effects were unlikely to have materially affected the pooled results. Nevertheless, because the number of included studies was relatively small, the presence of minor small-study effects cannot be completely dismissed.

## 4. Discussion

Contrast-enhanced CT remains the gold standard for diagnosing acute appendicitis; however, recent studies indicate that non-contrast CT (NCCT) demonstrates similar diagnostic accuracy and may serve as a reliable option for patients who cannot receive contrast agents [44,45,46,47]. Nevertheless, in patients with contraindications to contrast media, such as renal impairment or allergic reactions, the diagnostic value of NCCT has remained a subject of debate when compared with contrast-enhanced CT [48,49,50,51,52].

This systematic review and meta-analysis evaluated the diagnostic accuracy of NCCT for acute appendicitis. The results showed that NCCT demonstrated moderate diagnostic performance, with a pooled sensitivity of 0.93 and specificity of 0.97. The area under the HSROC curve (AUC) was 0.89 (95% CI, 0.83–0.96), demonstrating moderate overall diagnostic ability for identifying acute appendicitis.

According to the meta-analysis conducted by Chen et al. (2023), contrast-enhanced CT demonstrated a pooled sensitivity of 0.95 (95% CI, 0.93–0.96) and a specificity of 0.94 (95% CI, 0.93–0.96) for diagnosing acute appendicitis [47]. Likewise, the Cochrane meta-analysis by Rud et al. (2019) reported that intravenous contrast–enhanced CT showed a pooled sensitivity of 0.96 (95% CI, 0.92–0.98) and a specificity of 0.93 (95% CI, 0.90–0.95) among adult patients with suspected acute appendicitis [4].

In the present study, NCCT demonstrated moderate overall diagnostic accuracy, with performance comparable to that of contrast-enhanced CT. Previous evidence supports these findings. Hlibczuk et al. (2010) [53], in a systematic review of seven studies involving 1060 patients, reported that NCCT achieved a pooled sensitivity of 92.7% and a specificity of 96.1% in diagnosing acute appendicitis. Similarly, Chen et al. (2023) analyzed eight studies with a total of 1602 patients and found a pooled sensitivity of 0.85 and specificity of 0.93, suggesting that although the sensitivity was slightly lower than that of contrast-enhanced CT, NCCT remains a reliable and clinically valuable diagnostic tool [47].

However, because previous studies have reported variations in the diagnostic performance of NCCT for acute appendicitis, the present meta-analysis included a larger sample comprising 11 studies and 1996 patients. The results consistently demonstrated the moderate diagnostic accuracy of NCCT, further supporting its role as a reliable and clinically valuable imaging modality for evaluating acute appendicitis.

NCCT can serve as a safe and practical imaging option for patients with suspected acute appendicitis, particularly in those with contraindications to iodinated contrast media or in emergency situations requiring rapid clinical decision-making. In addition to avoiding contrast-related risks, NCCT offers several practical advantages, including shorter examination time, lower overall cost, and reduced delays associated with premedication in patients with contrast allergies or impaired renal function. Furthermore, because NCCT is performed as a single-phase examination without additional contrast-enhanced acquisitions, it inherently results in lower cumulative radiation exposure than multiphase contrast-enhanced CT [54,55]. These advantages may be especially relevant in younger populations or in individuals who require repeated imaging. Prior studies have also reported that NCCT protocols can streamline workflow and reduce overall imaging time in busy emergency department settings [56,57,58,59].

Several factors may account for the observed diagnostic performance of NCCT. With the widespread adoption of modern multidetector CT (MDCT) technology, high-resolution imaging has become routinely available, allowing precise visualization of subtle anatomic details. These advances enable radiologists to clearly identify characteristic findings of acute appendicitis, such as peri-appendiceal fat stranding, luminal distension, and mural thickening of the appendix, even without the use of intravenous contrast. Collectively, these imaging features provide sufficient diagnostic confidence when evaluating inflammatory changes of the appendix [49,60,61].

Several limitations should be taken into account when interpreting the findings of this meta-analysis. First, substantial heterogeneity was observed across the included studies, particularly with respect to specificity. This variation likely reflects differences in patient characteristics, CT acquisition techniques, diagnostic thresholds, and reference standards used in each study. In particular, the diagnostic criteria used for identifying acute appendicitis on non-contrast CT varied across the included studies. Most studies relied on common primary findings such as appendiceal enlargement, mural thickening, and peri-appendiceal fat stranding, whereas others incorporated secondary features including appendicolith, ill-defined mural margins, mural stratification, or peri-appendiceal fluid collections. In some earlier studies, a diagnosis was made even when the appendix was not clearly visualized, provided that surrounding inflammatory changes were present. Such inconsistencies in diagnostic thresholds may have contributed to the heterogeneity observed in specificity and should be taken into account when interpreting the pooled estimates. Second, methodological reporting was insufficient in several publications. Important details such as CT acquisition protocols, reader experience, and the use of blinding were inconsistently described, limiting the interpretability of the individual study results. Third, the inclusion criteria varied considerably among studies, which may have introduced selection bias, as also reflected in the QUADAS-2 assessment. Fourth, the majority of included studies did not provide a direct comparison between non-contrast CT and contrast-enhanced CT. As a result, any comparison between the two modalities in this review relies on indirect evidence and should be interpreted with caution. It should also be noted that our study did not perform a direct head-to-head comparative meta-analysis between NCCT and contrast-enhanced CT; therefore, the comparison presented here is based entirely on previously published pooled estimates. Finally, although Egger’s regression test (*p* = 0.079) did not indicate statistically significant publication bias, the relatively small number of included studies means that small-study effects cannot be completely excluded.

This study reinforces the clinical value of performing NCCT as an initial imaging modality in patients with suspected acute appendicitis who have renal impairment or a history of contrast allergy. In such cases, prioritizing NCCT over the administration of premedication or alternative contrast agents may provide a faster and safer diagnostic pathway, facilitating timely and accurate decision making in emergency settings. Moreover, NCCT offers additional benefits such as shorter examination time, lower cost, and avoidance of contrast-related complications, making it particularly useful in emergency departments or resource-limited environments. With the growing emphasis on minimizing unnecessary contrast exposure in patients at risk of renal dysfunction or hypersensitivity reactions, the present findings further support the broader clinical adoption of NCCT protocols as a safe, efficient, and reliable diagnostic option for evaluating acute appendicitis.

Most of the non-contrast CT studies included in this review were conducted between 1999 and 2007, reflecting the developmental stage of CT imaging for suspected appendicitis at that time. In the late 1990s and early 2000s, CT technology transitioned from single-slice helical scanners to multidetector CT, leading to marked improvements in image resolution and scanning speed. These advances prompted active investigation of both contrast-enhanced and non-contrast CT for the evaluation of acute appendicitis. Because many of the eligible studies were performed during the period when single-slice helical CT was predominantly used, the pooled estimates in this meta-analysis primarily represent the diagnostic performance of earlier CT systems and may not fully capture the capabilities of contemporary multidetector CT.

Moreover, many of the included studies were conducted more than a decade ago, and the evolution of CT hardware, imaging protocols, and diagnostic criteria over time may have influenced the aggregated results. Considering the substantial technological progress in CT over the past decade, further research using current multidetector CT platforms is warranted. Prospective multicenter studies that apply standardized imaging protocols and clearly defined diagnostic criteria would help reduce methodological variability, improve comparability across studies, and provide more reliable and up-to-date evidence regarding the diagnostic value of non-contrast CT in acute appendicitis. Future studies should also report diagnostic performance across relevant patient subgroups to strengthen the evidence base.

## 5. Conclusions

This meta-analysis shows that non-contrast computed tomography (NCCT) offers moderate diagnostic accuracy for evaluating acute appendicitis. NCCT remains a rapid, safe, and practical imaging option, particularly for patients with renal dysfunction, contrast hypersensitivity, or in emergency settings where timely decision-making is essential. Although the diagnostic performance of NCCT appears comparable to that of contrast-enhanced CT, this conclusion is based on indirect comparisons rather than direct head-to-head evidence. Further studies using contemporary CT technologies and standardized protocols are needed to more clearly define the relative diagnostic value of NCCT in current clinical practice.

## Figures and Tables

**Table 1:** Summary of the characteristics of the included studies.

Year	Author	Country	Sample Size	Age (Mean or Range, Y)	Study Design	TP	FP	FN	TN	Sensitivity (%)	Specificity (%)	PPV (%)	NPV (%)	Accuracy (%)
1999	Lane	USA	300	35 (4–89)	Prospective	110	4	5	181	96.0	99.0	96.5	97.3	97.0
1999	Stacher	Austria	56	36 (18–82)	Prospective	21	0	1	34	95.5	100.0	100.0	97.1	98.2
2000	Peck	USA	364	25 (2–92)	Retrospective	103	1	8	252	92.8	99.6	99.0	96.9	97.5
2000	Horton	USA	49	18–65	Retro + Prospective	36	0	1	12	97.3	100.0	100.0	92.3	98.0
2002	Morris	USA	129	Mean 35 (16–95)	Retrospective	38	8	1	82	97.4	91.1	82.6	98.8	93.0
2002	Ege	Turkey	296	24.7 (16–69)	Retrospective	104	3	4	185	96.3	98.4	97.2	97.9	97.6
2004	in’t Hof	Netherlands	103	Median 36 (16–82)	Prospective	83	0	4	16	95.4	100.0	100.0	80.0	96.1
2005	Keyzer	Belgium	94	Mean 38 (16–81)	Prospective	26	5	4	59	86.7	92.2	83.9	93.7	90.4
2006	Ashraf	Pakistan	61	N/A	Prospective	21	0	2	38	91.3	100.0	100.0	95.0	96.7
2007	Tamburrini	Italy	404	38 (18–86)	Retrospective	73	13	8	310	90.1	96.0	84.8	97.4	94.8
2021	Eurboonyanun	Thailand	140	52 ± 18 (Mean ± SD)	Retrospective	46	11	11	72	80.7	86.7	80.7	86.7	84.3

TP = true positive, FP = false positive, TN = true negative, FN = false negative, PPV = Positive Predictive Value, NPV = Negative Predictive Value, N/A = not available.

## Data Availability

All data generated or analyzed during this study are included in this published article and its Supplementary Materials.

## Supplementary Materials

The following supporting information can be downloaded at: https://www.mdpi.com/article/10.3390/medicina61122163/s1, Table S1: Detailed Search Strategies for Each Database; Table S2: The QUADAS-2 Tool for the Quality Assessment of Diagnostic Accuracy Studies; Table S3: QUADAS-2 Numerical Summary.

## Abbreviations

The following abbreviations are used in this manuscript:

CT	Computed Tomography
NCCT	Non-Contrast Computed Tomography
US	Ultrasound
PRISMA	Preferred Reporting Items for Systematic Reviews and Meta-Analyses
PubMed	US National Library of Medicine’s database of biomedical literature
Ovid MEDLINE	Online database of biomedical articles
EMBASE	Online database of biomedical articles
Cochrane Library	Collection of databases of systematic reviews and other evidence
QUADAS-2	Quality Assessment of Diagnostic Accuracy Studies-Second Edition
PPV	Positive Predictive Value
NPV	Negative Predictive Value
SROC	Summary Receiver Operating Characteristic Curve
HSROC	Hierarchical Summary Receiver Operating Characteristic Curve
AUC	Area Under the Curve
CI	Confidence Interval
