# Supplementary material for: Diagnostic Accuracy of Non-Contrast CT for Acute Appendicitis in the Emergency Department: A Systematic Review and Meta-Analysis
Source: Medicina (Kaunas). 2025 Dec 4;61(12):2163. doi: 10.3390/medicina61122163 (PMC12734481; doi:10.3390/medicina61122163)
Supplement: Supplementary file 1 [file medicina-61-02163-s001.zip › medicina-3998488-supplementary.pdf]

Supplementary Materials: Table S1. Detailed Search Strategies for Each Database.

Database	Search Terms Used	Filters Applied
PubMed	(“computed tomography”[MeSH Terms] OR “CT” OR “non-contrast CT” OR “unenhanced CT” OR “non-enhanced CT” OR “helical CT” OR “spiral CT”) AND (“appendicitis”[MeSH Terms] OR “appendix”)	English; Human; Up to June 2025
Ovid MEDLINE	(computed tomography OR CT OR non-contrast CT OR unenhanced CT OR non-enhanced CT OR helical CT OR spiral CT).mp. AND (appendicitis OR appendix).mp.	English; Human; Up to June 2025
EMBASE	(‘computed tomography’/exp OR ‘CT’ OR ‘non-contrast CT’ OR ‘unenhanced CT’ OR ‘non-enhanced CT’ OR ‘helical CT’ OR ‘spiral CT’) AND (‘appendicitis’/exp OR ‘appendix’)	English; Human; Up to June 2025
Cochrane Library	(computed tomography OR CT OR non-contrast CT OR unenhanced CT OR non-enhanced CT OR helical CT OR spiral CT) in Title Abstract Keyword AND (appendicitis OR appendix) in Title Abstract Keyword	No date restriction; English
Google Scholar	allintitle: (“CT” OR “computed tomography” OR “non-contrast CT” OR “unenhanced CT” OR “non-enhanced CT” OR “helical CT” OR “spiral CT”) AND (appendicitis OR appendix)	First 100 results screened manually

Supplementary Materials: Table S2. The QUADAS-2 Tool for the Quality Assessment of Diagnostic Accuracy Studies.

	Item	Yes	No	Unclear
1.	Was the spectrum of patients representative of the patients who will receive the test in practice?			
2.	Were selection criteria clearly described?			
3.	Is the reference standard likely to correctly classify the target condition?			
4.	Is the time period between reference standard and index test short enough to be reasonably sure that the target condition did not change between the two tests?			
5.	Did the whole sample or a random selection of the sample, receive verification using a reference standard of diagnosis?			
6.	Did patients receive the same reference standard regardless of the index test result?			
7.	Was the reference standard independent of the index test (i.e., the index test did not form part of the reference standard)?			
8.	Was the execution of the index test described in sufficient detail to permit replication of the test?			
9.	Was the execution of the reference standard described in sufficient detail to permit its replication?			
10.	Were the index test results interpreted without knowledge of the results of the reference standard?			
11.	Were the reference standard results interpreted without knowledge of the results of the index test?			
12.	Were the same clinical data available when test results were interpreted as would be available when the test is used in practice?			
13.	Were uninterpretable/ intermediate test results reported?			
14.	Were withdrawals from the study explained?			

Supplementary Materials: Table S3. The QUADAS-2 Numerical Summary.

Domain	Low (%)	Unclear (%)	High (%)
Patient Selection	36%	55%	9%
Index Test	27%	73%	0%
Reference Standard	45%	55%	0%
Flow and Timing	18%	82%	0%
